# Glutathione in Cellular Redox Homeostasis: Association with the Excitatory Amino Acid Carrier 1 (EAAC1)

**DOI:** 10.3390/molecules20058742

**Published:** 2015-05-14

**Authors:** Koji Aoyama, Toshio Nakaki

**Affiliations:** Department of Pharmacology, Teikyo University School of Medicine, 2-11-1 Kaga, Itabashi, Tokyo 173-8605, Japan; E-Mail: kaoyama@med.teikyo-u.ac.jp

**Keywords:** glutathione, oxidative stress, EAAC1, neurodegeneration

## Abstract

Reactive oxygen species (ROS) are by-products of the cellular metabolism of oxygen consumption, produced mainly in the mitochondria. ROS are known to be highly reactive ions or free radicals containing oxygen that impair redox homeostasis and cellular functions, leading to cell death. Under physiological conditions, a variety of antioxidant systems scavenge ROS to maintain the intracellular redox homeostasis and normal cellular functions. This review focuses on the antioxidant system’s roles in maintaining redox homeostasis. Especially, glutathione (GSH) is the most important thiol-containing molecule, as it functions as a redox buffer, antioxidant, and enzyme cofactor against oxidative stress. In the brain, dysfunction of GSH synthesis leading to GSH depletion exacerbates oxidative stress, which is linked to a pathogenesis of aging-related neurodegenerative diseases. Excitatory amino acid carrier 1 (EAAC1) plays a pivotal role in neuronal GSH synthesis. The regulatory mechanism of EAAC1 is also discussed.

## 1. Introduction

More than 50 years ago, the “free radical theory of aging” suggested that endogenous oxygen radicals generated in cells were a risk factor for aging-related diseases, and a thiol compound such as cysteine was expected to slow the aging process [1]. Subsequent studies have shown the importance of disequilibrium in cellular reduction-oxidation status, called “redox” status, as a cause of oxidative stress [2]. Accumulated insults from oxidative stress damage cellular functions, especially in vulnerable tissues. In the present review, we focus on the regulation of cellular redox homeostasis especially in the central nervous system (CNS).

## 2. Glutathione as a Redox Buffer

Thiols, also called sulfhydryls, play pivotal roles in cellular redox homeostasis. There are four thiol-containing amino acids—cysteine, methionine, homocysteine, and taurine—but cysteine and methionine are the major thiol-containing amino acids involved in cellular metabolism in mammals [3]. Glutathione (GSH) is a cysteine-containing tripeptide, which is the most abundant nonprotein thiol in cells [4]. GSH is composed of glutamate, cysteine, and glycine and is synthesized intracellularly via two reactions catalyzed by γ-glutamylcysteine ligase (GCL) and GSH synthetase (GS) [5,6]. The reaction of glutamate with cysteine is catalyzed by GCL to produce a dipeptide, γ-glutamylcysteine, which then reacts with glycine catalyzed by GS to produce GSH. In this process, cysteine, but not glutamate or glycine, is the rate-limiting substrate because intracellular concentrations of both glutamate and glycine are higher than those of the K_m_ values of the reactions [6]. GSH is present abundantly in the cells at millimolar concentrations in the liver > kidney > spleen > small intestine > brain > pancreas > lung > heart > muscle [7]. In the liver, kidney, intestine, and pancreas, cysteine is also supplied via the transsulfuration pathway, which converts methionine through homocysteine to cysteine [3,8].

GSH is considered the main redox buffer in a cell because of the large amount of reducing equivalents supplied by GSH [9]. The sulfhydryl residues of GSH molecules are easily oxidized to form GSH disulfide (GSSG), which is then reduced back to GSH by the reaction with GSH reductase (GR). The intracellular thiol redox status is described as the ratio of reduced to oxidized forms, GSH/GSSG, showing 100 or more at the steady state but decreasing to 10 or less under oxidative stress conditions [10,11]. Intracellular redox changes affect cell signaling, gene transcription, gene translation, cell proliferation, and cell death [12,13,14,15,16]. Proteins contain abundant sulfhydryl residues derived from their cysteine side-chains, which comprises up to 3% of the total amino acids in human [17]. Residues of cysteine, as well as those of methionine, tryptophan, and tyrosine, are prone to oxidative modification. Oxidation in the intracellular redox environment induces irreversible protein thiol oxidation and thereby alters protein functions as enzymes, receptors, and transporters [18]. Under oxidative stress conditions, GSH can reversibly form mixed disulfide bonds between protein thiols (*S*-glutathionylation) to prevent protein oxidation [19].

The redox state is more oxidative in the endoplasmic reticulum (ER) than in the cytosol; the GSH/GSSG ratio in the ER ranges from 1 to 3 [20]. This ratio is preferable for the folding of disulfide bond-containing proteins in the ER [20,21]. An initial observation suggested that the ER preferentially transports GSSG rather than GSH into the lumen in order to establish an oxidative environment [20]. However, subsequent studies showed the selective transport of GSH across the ER membrane [22,23]. Indeed, less than 50% of total GSH (GSH + GSSG) was free in the ER, while the remainder was found as mixed disulfides with proteins [21]. Although the significance of the low GSH/GSSG ratio is still elusive, protein disulfide formation with GSH in the ER might play an important role in protecting protein functions against oxidative stress.

## 3. Antioxidant Defense System

**Figure 1:**
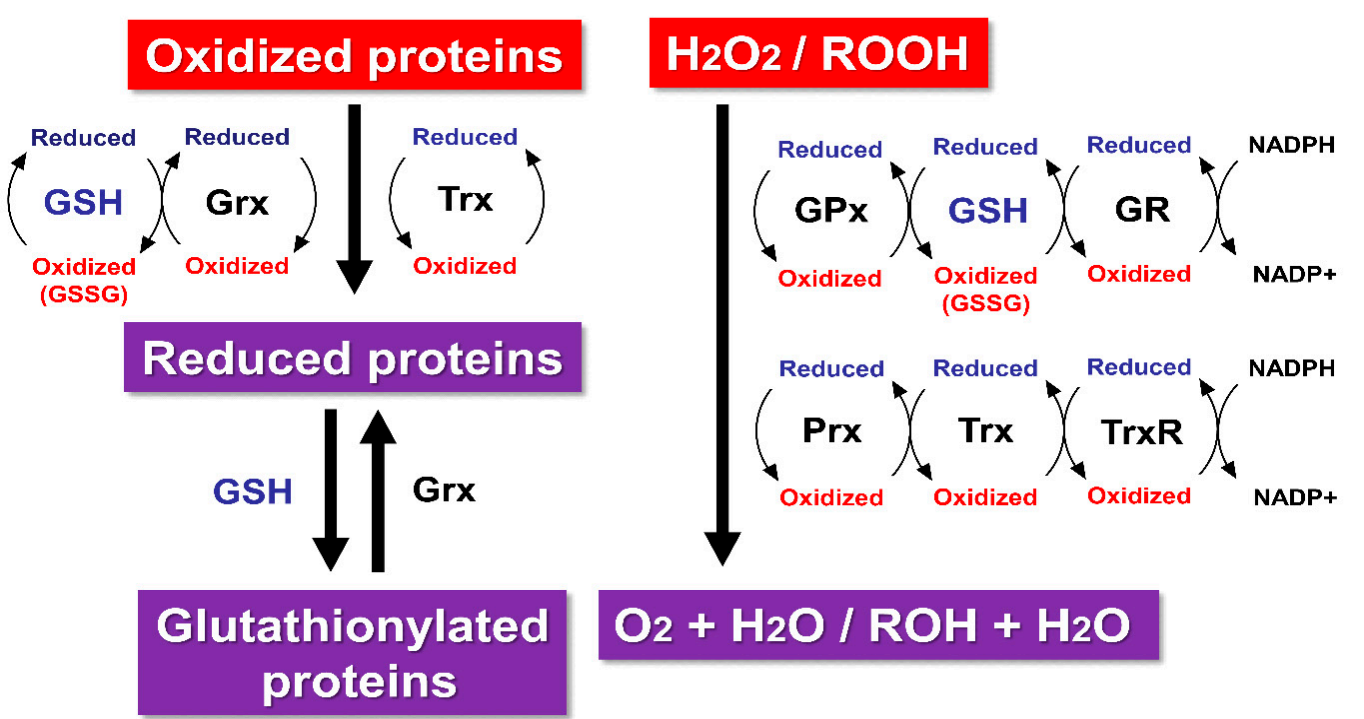
Regulation of the redox homeostasis by glutathione (GSH), thioredoxin (Trx), and glutaredoxin (Grx) systems. Hydrogen peroxide (H_2_O_2_) and hydroperoxides (ROOH) are catalyzed by GSH peroxidase (GPx) or peroxiredoxin (Prx) to alcohols (ROH) and water. The oxidized form of Trx is reduced back by the reaction with Trx reductase (TrxR), while that of Grx is reduced back by GSH. An oxidized GSH (GSSG) is reduced back to two GSH molecules by the reaction of GSH reductase (GR). Both Trx and Grx reduce protein disulfides. Grx also catalyzes protein deglutathionylation.

The human brain requires ~20% of the oxygen consumed by the body, even though it occupies only 2% of body weight. It contains high levels of unsaturated fatty acids, which would be targets for oxidative stress, but relatively low levels of antioxidants and related enzymes [5]. Reactive oxygen species (ROS) are endogenously produced from mitochondria, cytochrome P450 metabolism, peroxisomes, and inflammatory cell activation [24,25,26]. Notably, mitochondria generate ATP from ADP as a cellular energy molecule via oxidative phosphorylation. Oxidative phosphorylation is coupled with an electron transport chain, also known as a respiratory chain, on the mitochondrial inner membrane to pump protons out of the matrix and into the intermembrane space. This electrical proton gradient generates proton-motive force to synthesize ATP. During electron transfer through the respiratory chain, mitochondria generate large portions of ROS, such as superoxide, hydroxyl radical, hydroperoxyl radical, and hydrogen peroxide (H_2_O_2_), into the matrix and the intermembrane space. Previous reports have suggested that 2% of the total oxygen consumption in the mitochondrial respiratory chain produces superoxide generating H_2_O_2_ [27]. However, a recent study showed that only about 0.15% of electron flow in mitochondria is converted to H_2_O_2_ under resting conditions [28]. Although the steady state concentration of superoxide is approximately 5- to 10-fold higher in the mitochondrial matrix than in the cytosol and the nucleus [29], the steady state concentrations of mitochondrial superoxide and H_2_O_2_ are estimated to be as low as about 10^−10^ M and 10^−9^~10^−8^ M, respectively [27,29]. These results are attributed to ROS scavenging by the antioxidant defense system in mitochondria to prevent H_2_O_2_ from leaking into the cytosol. Superoxide is catalyzed to H_2_O_2_ by manganese superoxide dismutase (Mn-SOD) or copper/zinc-SOD (Cu/Zn-SOD). The mitochondrial matrix contains higher levels of Mn-SOD (1.1 × 10^−5^ M) compared to other compartments in a cell [30]. Approximately 90% of Cu/Zn-SOD (2.4 × 10^−5^ M) was found in the cytosol, and ~3% of that was found in the mitochondrial intermembrane space [30]. Superoxide generated in the mitochondrial matrix reacts with Mn-SOD, while that released into the intermembrane space or cytosol reacts with Cu/Zn-SOD. Generally, H_2_O_2_ is toxic to eukaryotic cells in the range of 0.1~1 × 10^−3^ M *in vitro*; however, such high concentrations of H_2_O_2_ are improbable under physiological conditions *in vivo* because H_2_O_2_ is then degraded to oxygen and water by the reaction with catalase, peroxiredoxin (Prx), or GSH peroxidase (GPx) (Figure 1).

Peroxisomes play an essential part in cellular fatty acid metabolism [31] via biochemical oxidations leading to both superoxide and H_2_O_2_ generation [32]. Peroxisomal H_2_O_2_ is metabolized mainly by catalase, a peroxisomal antioxidant [33]. One molecule of catalase can convert approximately 6 million molecules of H_2_O_2_ to oxygen and water per minute [34]. Catalase has a high Km value to H_2_O_2_, while GPx as a low one [35]. Catalase can react with H_2_O_2_ but not with other hydroperoxides, while Prx and GPx can react with both [36].

Prx and GPx have peroxidase activity in thioredoxin (Trx)- and glutaredoxin (Grx)-dependent manners, respectively [37] (Figure 1). After the reaction with H_2_O_2_, the oxidized Prx is reduced back by the reaction with Trx, while the oxidized GPx is reduced back mainly by the reaction with GSH [38]. In mammals, Trx and Grx isoforms have been characterized as cytosolic Trx1 and Grx1, and as mitochondrial Trx2 and Grx2 [37]. Trx and Grx are endogenous antioxidants that play important roles as electron donors in the cellular redox homeostasis and are also the primary reductants of disulfide bonds of intracellular proteins to protect cells against oxidative stress or apoptosis [37,39]. Subsequently, the oxidized form of Trx is reduced back by the reaction with Trx reductase (TrxR), while that of Grx is reduced back by GSH. Although GSH is then oxidized to GSSG, GSH reductase (GR) can regenerate GSH from GSSG. The reaction of GR with GSSG is regulated by nicotinamide adenine dinucleotide phosphate (NADPH), which is produced by the reaction of glucose-6-phosphate dehydrogenase with NADP^+^ [40]. Grx also catalyzes both the formation and reduction of glutathionylated proteins, although the latter is the main function in general [41,42]. TrxR and GR are NADPH-dependent flavoenzymes that transfer electrons to oxidized Trx and GSSG, respectively. TrxR has three isoforms: cytosolic TrxR1, mitochondrial TrxR2, and testis-specific Trx/GSH reductase [37]. It reacts not only with oxidized Trx but also with lipid hydroperoxides and H_2_O_2_ [37]. Prx is a Trx-dependent peroxidase with six isoforms, which is localized to cytosol (Prx I, II, V, and VI) mitochondria (Prx III and V), ER (Prx IV), and microsome (Prx V) [43]. Prx is kept in a reduced form as a peroxidase enzyme that receives electrons from NADPH by coupling with Trx and TrxR [44]. GPx has eight isoforms identified as selenoproteins with a selenocysteine in the catalytic center (GPx1-4 and 6) or nonselenoproteins (GPx 5, 7, and 8) [38]. Among the seleno-containing isoforms, GPx1-4 are found in mammals and GPx6 only in humans [38]. GPx1 is the predominant isoform, expressing ubiquitously in the tissues and localizing mainly to the cytosol; it is also present in the mitochondrial matrix [45], although the estimated concentration of mitochondrial GPx is lower (1.17 × 10^−6^ M) than that of cytosolic GPx (5.8 × 10^−6^ M) [29,46]. It seems that GPx is the leading H_2_O_2_ scavenger in mitochondria [29]. In this antioxidant defense system, superoxide release is undetectable in intact mitochondria [47]. However, mitochondrial dysfunction leads to increased production of both superoxide and H_2_O_2_ [48,49]. The ratio of steady state concentration of mitochondrial superoxide to H_2_O_2_, represented by [H_2_O_2_]/[superoxide], is under 100, whereas that in the cytosol is estimated to be 1,000 [29].

Considering the importance of cellular redox status, dysregulation in the redox status by GSH under pathological conditions would be involved in some human diseases such as hemolytic anemia, human immunodeficiency virus infection/acquired immune deficiency syndrome, liver disease, and cystic fibrosis [50]. Especially, GSH seems to be important in the brain because inborn errors in GSH metabolism induce neurological symptoms such as ataxia, mental retardation, seizures, spasticity, hearing loss, motor impairment or tremor *etc.* [51]. Moreover, several age-related neurodegenerative diseases have been involved in disorders of GSH metabolism [6]. Many lines of evidence suggest mitochondrial involvement in the pathogenesis of aging-related neurodegenerative diseases [52]. There have been reports of abnormal protein expression of Prx isoforms in the brains of neurodegenerative diseases such as Alzheimer’s disease (AD), frontotemporal dementia, Parkinson’s disease (PD), amyotrophic lateral sclerosis (ALS), and Huntington’s disease (HD) [53,54,55,56,57]. The Trx and Grx systems are also involved in these neurodegenerative diseases [58,59,60,61]. AD patients showed reduced blood antioxidant enzyme activities, including those of SOD, catalase, GPx, and GR [62]. *GPx1* polymorphism showing a 70% decrease in enzyme activity was identified as a positive risk factor for AD [63]. Similarly, GPx activity was significantly reduced in the substantia nigra of PD patients [64]. In addition, progressive supranuclear palsy is another aging-related neurodegenerative disease showing the involvement of oxidative stress and GSH depletion in the brain [65,66]. In the CNS of progressive supranuclear palsy patients, GPx enzymatic activity is thought to be decreased by conjugation with a lipid peroxidation product, 4-hydroxy-2-nonenal [67,68]. Perturbations of cellular redox status would be closely linked to the disruption of the antioxidant systems leading to neurodegeneration.

## 4. ROS/RNS Generation

Excessive ROS generation leading to oxidative stress has been implicated in the progression of some neurodegenerative diseases including AD, PD, ALS, and HD [52,69,70,71]. Especially, the production of reactive nitrogen species (RNS), which are nitric oxide (NO)-derived oxidants, has been involved in the pathogenesis of these neurodegenerative diseases [72,73,74].

NO is synthesized from L-arginine by the reaction with NO synthases (NOS) [75]. Three types of NOS have been identified: neuronal NOS (nNOS, type I), inducible NOS (iNOS, type II), and endothelial NOS (eNOS, type III) [75]. Glutamate, an excitatory neurotransmitter, activates *N*-methyl-d-aspartate (NMDA) receptors to open a channel permeable to Ca^2+^, leading to Ca^2+^/calmodulin-dependent activations of both nNOS and eNOS [76], but not iNOS, which is mainly regulated by NFkB activation [77]. NO can diffuse widely (~400 μm) [78] and react with superoxide to form peroxynitrite, which is a potent oxidant [73]. Peroxynitrite can damage DNA, membrane lipids, mitochondria, and proteins and induce cell death via a necrotic or apoptotic mechanism depending on production rates, endogenous antioxidant levels, and exposure time [74]. Peroxynitrite is generated at the site of superoxide production because the half-life of superoxide (10^−6^ s) is shorter than that of NO (~1 s) [73]. The rates of peroxynitrite production *in vivo* have been estimated to be as high as 50–100 μM per min [74]. The half-life of peroxynitrite is approximately 10 ms, which is enough to cross the cell membrane and influence surrounding cells at physiological pH [79,80]. No enzyme is necessary to form peroxynitrite, and the rate of superoxide reacting with NO (~1 × 10^10^ M^−1^s^−1^) is faster than that reacting with SOD (~2 × 10^9^ M^−1^s^−1^) [73,74]. Basal NO levels are below 10^−8^ M, which is too low to effectively compete with SOD [81], although it will increase 100-fold under pathological conditions [82,83]. Furthermore, the reaction rate of superoxide and NO is elevated in a synergistic manner; *i.e.*, the production of both superoxide and NO is increased 1,000-fold, which will increase the formation of peroxynitrite by 1,000,000-fold [74].

Many biomolecules, including tyrosine, tryptophan, guanine, cysteine, lysine, methionine and histidine residues, DNA, and fatty acids, are oxidized and/or nitrated by peroxynitrite-derived radicals [74,84]. These biological reactions induce the inhibition (and sometimes the activation) of enzymes, receptors, transporters, and membrane channels, as well as protein aggregation, impairment of cellular signaling, mitochondrial dysfunction, DNA injury, and lipid peroxidation [74,85]. Particularly in mitochondria, peroxynitrite can cause inactivation of the electron transport chain complex I, II and V, leading to superoxide and H_2_O_2_ generation [86]. Moreover, peroxynitrite can inhibit Mn-SOD activity by the nitration of a critical tyrosine-34 residue, leading to the exacerbation of mitochondrial injury [87,88]. Nitrated Mn-SOD levels were increased in the CNS of AD, PD, and ALS patients [72].

H_2_O_2_ reacts with Fe^2+^ (Fenton’s reaction) to form a highly oxidizing intramolecular radical, hydroxyl radical [89]. The rate constant of Fenton’s reaction is about 1.2–1.3 M^−1^s^−1^ [29], which is much slower than that of peroxynitrite formation (6.7 × 10^9^ M^−1^s^−1^) [81]. Consequently, hydroxyl radical formation needs transition metals near a critical site to inactivate the biological target [81]. Hydroxyl radical is also produced by peroxynitrite decomposition [74,81], but this reaction is slow in biological systems [74]. Hydroxyl radical scavengers did not reduce peroxynitrite-induced cytotoxicity [90], suggesting that peroxynitrite-induced hydroxyl radical formation has a minor role in the toxicity of peroxynitrite. Hydroxyl radical can diffuse only about the diameter of a typical protein, which is limited to much less than that of peroxynitrite [81,91]. However, hydroxyl radical is a powerful oxidant, which attacks any organic molecules [81,91]. Amyloid β and α-synuclein, abnormal aggregated proteins in AD and PD, respectively, can both generate hydroxyl radical after incubation with Fe^2+^ *in vitro* [92]. Hydroxyl radical generation via Fenton’s reaction might be involved in the progression of these neurodegenerative diseases.

GSH directly reacts with superoxide, NO, peroxynitrite, and hydroxyl radical. The ability of GSH to scavenge superoxide varies among the published reports, with rate constants ranging from 10^2^ to 10^5^ M^−1^s^−1^ [93]. The rate constants of NO, peroxynitrite, and hydroxyl radical with GSH are ~3 × 10^5^ M^−1^s^−1^, ~281 M^−1^s^−1^, and 1.3 × 10^10^ M^−1^s^−1^, respectively [93,94,95]. Considering the high intracellular concentrations, GSH acts as a potent antioxidant against a variety of ROS, while GSH depletion is caused by increased oxidative stress and/or decreased GSH synthesis, especially in neurons, which are more vulnerable to ROS than are glial cells [5].

## 5. Glutathione as a Regulator of Redox Signal Transduction

Protein *S*-glutathionylation is a reversible post-translational modification not only for protection of cysteine residues from irreversible oxidation under oxidative stress conditions but also for transduction of redox signaling by changing structure/function of various target proteins, even in intact cellular system [96,97]. Like protein phosphorylation, the *S*-glutathionylation modulates enzyme activities, DNA binding by transcription factors, and protein stability [17,96,98]. The modifications of protein thiols in cysteine residues can alter protein functions because many proteins contain cysteine residues in their active sites or functional motifs [99,100]. More than 2200 target sites have been identified for *S*-glutathionylation, which is involved in cancer migration, cell death and survival, energy metabolism and glycolysis, as well as protein folding and degradation [101,102,103]. A number of papers have been published in different diseases showing abnormal protein *S*-glutathionylation as a potential biomarker [17]. For more precise information regarding protein *S*-glutathionylation, readers are referred to other reviews [96,99,102].

## 6. EAAC1 Dysfunction Leading to Neurodegeneration

Excitatory amino acid transporters (EAATs) regulate glutamatergic signaling via glutamate uptake from synaptic clefts into the cells [104]. Among the five EAAT isoforms, EAAT1-3 are the most widely expressed in the brain. EAAT1 (glutamate-aspartate transporter, GLAST) and EAAT2 (glutamate transporter-1, GLT-1) are expressed in glial cells and involved mainly in synaptic glutamate clearance, while EAAT3 (excitatory amino acid carrier 1, EAAC1) is expressed in mature neurons and involved in cysteine uptake rather than in glutamate clearance in the brain. Indeed, the downexpression of GLAST or GLT-1 increased extracellular glutamate levels in the CNS, while that of EAAC1 did not affect the levels [105]. Moreover, when arginine 447, a residue conserved in all EAATs, of EAAC1 is replaced by cysteine, glutamate transport is abolished but cysteine transport remains intact [106]. Mature neurons utilize extracellular cysteine for GSH synthesis, while astrocytes utilize cystine, which is formed by oxidation of two cysteines with a disulfide bond. Intracellular cysteine levels are the rate-limiting substrate for GSH synthesis [107]. Therefore, the cysteine transport system via EAAC1 is considered key for neuronal GSH synthesis. EAAC1-deficient mice showed age-dependent brain atrophy, learning/memory dysfunction, and reduced brain GSH levels [108]. These results indicate that the cysteine transport system of EAAC1 is a notable feature, and one that is independent of the other EAATs, for neuronal GSH synthesis. However, previous studies showed that EAATs are vulnerable to oxidative stress, leading to impaired transport function by peroxynitrite or H_2_O_2_ [85]. Previous studies also have demonstrated that oxidative stress reduced neuronal cysteine uptake via EAAC1 dysfunction, leading to impaired GSH synthesis in the mouse midbrain [109]. EAAC1-deficient mice showed age-dependent loss of dopaminergic neurons in the substantia nigra and increased oxidative stress [110]. Neuronal cysteine uptake by EAAC1 was inhibited by soluble amyloid β (Aβ) oligomers *in vitro* [111], and aberrant EAAC1 accumulations were found in the hippocampal neurons of AD patients [112]. In an *in vitro* study, cysteine uptake inhibition leading to GSH depletion via EAAC1 dysfunction was found in neurons from a mouse HD model that have human *huntingtin* exon1 with 140 *CAG* repeats inserted [113]. Oxidative stress causes EAAC1 dysfunction leading to neuronal GSH depletion, which enhances oxidative stress more. Aging is considered a precipitating factor for both increased oxidative stress [114] and decreased GSH levels in the brain [115]. Brain GSH depletion is considered to precede the clinical progression of age-related neurodegenerative diseases [69,116,117]. Although further clinical evidences are still needed for elucidating the precise mechanism, it is considered plausible that EAAC1 dysfunction leading to GSH depletion is closely involved in neurodegenerative diseases.

## 7. Regulation of EAAC1

**Figure 2:**
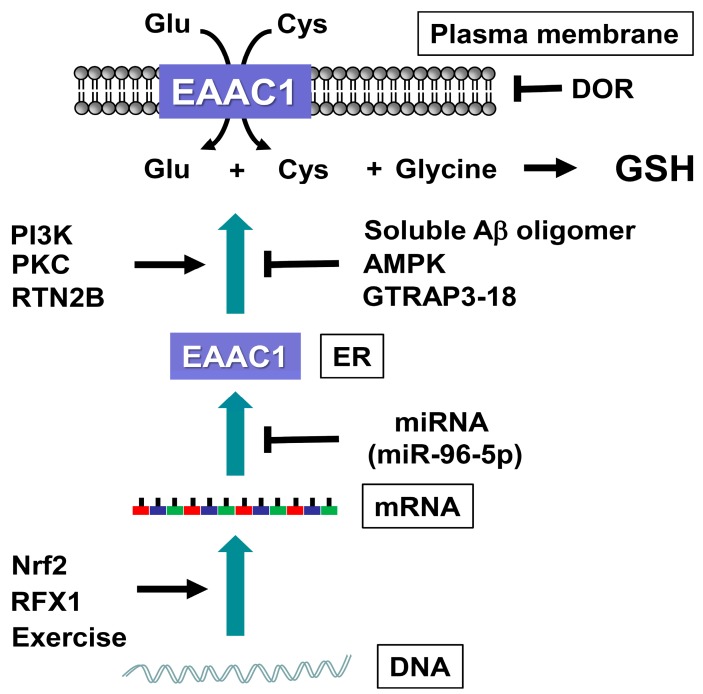
Regulatory mechanisms of EAAC1, which is a neuronal transporter for cysteine (Cys) and glutamate (Glu) uptake for glutathione (GSH) synthesis. Stimulatory (*arrow*) and inhibitory (˫) regulations for EAAC1 activity. The abbreviations are as follows: AMP-activated protein kinase (AMPK), amyloid β (Aβ), endoplasmic reticulum (ER), excitatory amino acid carrier 1 (EAAC1), δ-opioid receptor (DOR), glutamate transport associated protein 3-18 (GTRAP3-18), nuclear factor erythroid 2-related factor 2 (Nrf2), phosphatidylinositol 3-kinase (PI3K), protein kinase C (PKC), regulatory factor X1 (RFX1).

Redox status of sulfhydryl residues in EAAC1 affects its transport properties [118,119]. Oxidative modification of cysteine residues in EAAC1 decreased, while the reduced modification increased the glutamate transport activity [118,119]. Redox modulation of sulfhydryl residues in EAAC1 might constitute an important physiological or pathological roles in the regulation of the transport activity [118,119]. EAAC1 is also regulated at different levels related to DNA transcription, RNA translation, and protein expression on the cell surface (Figure 2). Glial EAATs, both GLAST and GLT-1, are predominantly expressed on the cell surface [120,121], while EAAC1 expresses only ~20% of the transporter on the cell surface [122]. EAAC1 is trafficked between intracellular compartments and the cell surface to change the transport activity [122,123,124]. Once stimulated by protein kinase C activation, EAAC1 expression doubles on the cell surface [122]. Phosphatidylinositol 3-kinase activation also increases cell surface expression of EAAC1, while AMP-activated protein kinase and glutamate transport-associated protein 3-18 (GTRAP3-18) inhibit EAAC1 translocation to the cell surface. RTN2B, a member of the reticulon family, enhances ER exit and the cell surface composition of EAAC1 [125]. Soluble Aβ oligomers inhibited EAAC1-mediated cysteine uptake and decreased intracellular GSH levels *in vitro*, even though mRNA expression of EAAC1 was reactively increased by treatment with soluble Aβ oligomers [111]. In our recent studies, GTRAP3-18-deficient mice showed increased EAAC1 expression on the cell surface, with increased neuronal GSH levels and neuroprotection against oxidative stress [126]. At forced motor/spatial learning and memory tests, GTRAP3-18-deficient mice performed better than age-matched wild-type mice [126]. The δ-opioid receptor, a G-protein coupled receptor, interacts directly with EAAC1 on the plasma membrane to reduce the glutamate transport activity [127]. For a detailed overview of this topic, we refer readers to our review articles [128,129,130]. EAAC1 protein expression is upregulated by transcriptional factors such as the nuclear factor erythroid 2-related factor 2 and the regulatory factor X1 [131,132]. The mRNA levels for EAAC1 in the rat hippocampus were upregulated after exercise [133]. On the other hand, EAAC1 protein expression is downregulated by miR-96-5p, which is a small noncoding RNA molecule, named microRNA, involved in the post-transcriptional regulation of gene expression. We have reported that cellular protection against ROS is time-dependently correlated with GSH rhythm, which is regulated by rhythmic miR-96-5p expression through the direct regulation of EAAC1 expression [134]. Upregulation of miR-96-5p decreased EAAC1 protein expression, leading to a reduction in GSH, while an miR-96-5p inhibitor increased the GSH level, leading to neuroprotection against oxidative stress via an increased level of EAAC1 [134]. The involvement of microRNA in neurodegenerative diseases is also discussed in our recent review [135]. These results indicate a potential strategy against neurodegeneration by increasing neuronal GSH via EAAC1 function.

## 8. Conclusions

The intracellular redox status is determined by a balance between oxidative stress and the antioxidant system. In the brain, a consecutive imbalance toward the pro-oxidant side impairs cellular functions, leading to neurodegeneration. GSH is the most abundant thiol-containing molecule in a cell; it regulates the cellular redox condition and plays a critical role in the anti-oxidant system. GSH depletion is involved in the pathogenesis of aging-related neurodegenerative diseases. Particularly, dysfunction of EAAC1, which is a neuronal transporter for cysteine and glutamate uptake, impairs neuronal GSH synthesis to cause GSH depletion in aging-related neurodegenerative diseases. Thus, the regulation of EAAC1 is critical for neuronal GSH synthesis to maintain cellular redox homeostasis. Upregulation of EAAC1 may be a potential strategy in neurodegenerative diseases.
